# Noncoding RNA-mediated molecular bases of chemotherapy resistance in hepatocellular carcinoma

**DOI:** 10.1186/s12935-022-02643-6

**Published:** 2022-08-09

**Authors:** Qikuan He, Pengyi Guo, Zhiyuan Bo, Haitao Yu, Jinhuan Yang, Yi Wang, Gang Chen

**Affiliations:** 1 Department of Hepatobiliary Surgery, The First Affiliated Hospital of Wenzhou Medical University, Wenzhou, 325035 Zhejiang China; 2 Key Laboratory of Diagnosis and Treatment of Severe Hepato-Pancreatic Diseases of Zhejiang Province, The First Affiliated Hospital of Wenzhou Medical University, Wenzhou, 325035 Zhejiang China; 3 Department of Cardiothoracic Surgery, Ningbo Yinzhou No. 2 Hospital, Ningbo, 315199 Zhejiang China; 4 Department of Epidemiology and Biostatistics, School of Public Health and Management, Wenzhou Medical University, Wenzhou, 325035 Zhejiang China

**Keywords:** HCC, Chemoresistance, CSCs, EMT, Autophagy, EVs, Immune surveillance, Ferroptosis, ncRNAs

## Abstract

Despite the significant progress in decreasing the occurrence and mortality of hepatocellular carcinoma (HCC), it remains a public health issue worldwide on the basis of its late presentation and tumor recurrence. To date, apart from surgical interventions, such as surgical resection, liver transplantation and locoregional ablation, current standard antitumor protocols include conventional cytotoxic chemotherapy. However, due to the high chemoresistance nature, most current therapeutic agents show dismal outcomes for this refractory malignancy, leading to disease relapse. Nevertheless, the molecular mechanisms involved in chemotherapy resistance remain systematically ambiguous. Herein, HCC is hierarchically characterized by the formation of primitive cancer stem cells (CSCs), progression of epithelial-mesenchymal transition (EMT), unbalanced autophagy, delivery of extracellular vesicles (EVs), escape of immune surveillance, disruption of ferroptosis, alteration of the tumor microenvironment and multidrug resistance-related signaling pathways that mediate the multiplicity and complexity of chemoresistance. Of note, anecdotal evidence has corroborated that noncoding RNAs (ncRNAs) extensively participate in the critical physiological processes mentioned above. Therefore, understanding the detailed regulatory bases that underlie ncRNA-mediated chemoresistance is expected to yield novel insights into HCC treatment. In the present review, a comprehensive summary of the latest progress in the investigation of chemotherapy resistance concerning ncRNAs will be elucidated to promote tailored individual treatment for HCC patients.

## Background

As one of the most typical and fatal malignancies worldwide, hepatocellular carcinoma (HCC) accounts for nearly 90% of all liver cancers and originates from primary hepatocytes under carcinogenic factors such as alcohol-related cirrhosis, hepatitis B & C virus and exposure to diverse dietary toxins, which causes over 600,000 deaths annually [1]. In the last several decades, the incidence and mortality of HCC have doubled due to its heterogeneity, with an unfavorable 5-year survival rate of no more than 10% among patients [2]. Despite advancements in the diagnosis and treatment of HCC, curative treatments such as surgical removal, locoregional ablation or liver transplantation are options for a minority of newly diagnosed individuals with relatively favorable prognoses [3]. Unfortunately, the majority of HCC cohorts are asymptomatic, and patients tend to be diagnosed at an advanced stage to receive palliative therapies, including systematic chemotherapy, radiotherapy and transcatheter arterial chemoembolization (TACE). In particular, systematic antitumor agents have played an increasingly promising role in the management of HCC with the development of multiple kinase inhibitors. However, progress in the field of conventional chemotherapy has stalled owing in a large part to chemoresistance that leaves patients with ineffective therapeutic choices and an unsatisfactory prognosis.

Pioneering studies have demonstrated that chemotherapy resistance presents a major hurdle in the current health care system, which in turn contributes to the tumorigenesis and progression of various carcinomas. To enforce strict regulation for advanced HCC, sorafenib is the first oral targeted medication approved by the FDA, and its therapeutic effects are accomplished through the inhibition of several kinases and signaling pathways, including the VEGFR kinase, EGFR kinase, PDGFR kinase, MAPK, NF-κB and STAT3 signaling pathways [4]. Specifically, sorafenib exhibits antitumor activity against HCC by inhibiting angiogenesis, suppressing oncogenic gene mutations and inducing tumor cell apoptosis. In addition, other kinase inhibitors and immune checkpoint inhibitors, such as sunitinib, erlotinib, lenvatinib, regorafenib, nivolumab, pembrolizumab, ipilimumab and tremelimumab, were subsequently established individually or in combination to improve curative efficacy [5, 6]. However, the overall survival (OS) rate of advanced HCC patients remains low, and less than one-third of them benefit from chemotherapy. Drug resistance is apparent within six months of initiating the treatment regimen [7]. Thus, further research is necessary to improve the outcome of such a devastating disease.

Currently, growing attention has been focused on the annotation of specific molecular mechanisms underlying chemoresistance. Accordingly, cancer stem cells (CSCs), epithelial-mesenchymal transition (EMT), autophagy, extracellular vesicles (EVs), immune evasion, ferroptosis, tumor microenvironment (TME) and multidrug resistance (MDR) relevant signaling pathways are considered pivotal determinants for drug resistance in HCC [8–12]. Notably, high-throughput sequencing and bioinformatics analysis have revealed the significance of noncoding RNAs (ncRNAs) in the mentioned physiological processes in a broad spectrum of cancers [13–16]. As the majority of transcriptional precursor RNAs, over 70% of the whole genome is transcribed into ncRNAs and regulates residual genes by employing epigenetic modification, transcriptional regulation, posttranscriptional modification and targeted protein translation. Nevertheless, until now, the crosstalk between ncRNAs and the molecular bases of chemoresistance has been poorly understood in HCC. Furthermore, strategies aimed at reversing ncRNA-mediated refractoriness are currently underway and represent a promising future that converts traditional agents into potent killers of cancerous cells. The current review provides an update of accessible information regarding ncRNA-mediated molecular mechanisms of drug resistance with a remarkable impact on the effectiveness of HCC therapy.

## The biogenesis and function of ncRNAs

ncRNAs refer to the prevalent clusters of RNA transcripts that cannot encode peptides or proteins. According to their classification, ncRNAs are divided into transfer RNAs (tRNAs), microRNAs (miRNAs), small interfering RNAs (siRNAs), circular RNAs (circRNAs), ribosomal RNAs (rRNAs), long noncoding RNAs (lncRNAs) and small nuclear RNAs (snRNAs). Alternatively, considered the “junk” of gene transcription, regulatory ncRNAs fall into three prevailing categories: miRNAs with a transcript length less than 200 nucleotides, lncRNAs with a transcript length exceeding 200 nucleotides and circRNAs with unique circular structures. Functionally, apart from directly operating on host gene expression, ncRNAs consistently tend to regulate cell processes in a coordinated network via internal interplay. Furthermore, as the central members, miRNAs have been demonstrated in numerous studies to be indispensably linked to lncRNAs, circRNAs and messenger RNAs (mRNAs) [17, 18]. Additionally, miRNAs and lncRNAs are biosynthesized via subsequent procedures with RNA polymerase II, and RNase III enzyme, ribonuclease III, among others. which is similar to the transcription process of mRNAs. Moreover, with stable molecular characteristics, circRNAs are mainly generated by the primary mRNA through a back splicing mechanism that is defined to reverse the downstream splicing site and connect to the upstream splicing site to form a closed circular molecule, which endows circRNAs with resistance to RNase R. Specifically, lncRNAs and circRNAs can act as competitive RNA or RNA sponges to bind miRNAs and restrict the regulation of mRNA targets. Nonetheless, the biogenesis and function of ncRNAs are complicated processes that require in-depth studies of regulatory details. Above all, given the distinct roles of ncRNAs in the regulation of HCC progression, we further describe the potential interaction between ncRNAs and chemoresistance-related features of HCC in the following sections.

## The involvement of ncRNAs in liver cancer stem cells (LCSCs)

Cancer stem cells (CSCs) represent a small proportion of pluripotent cancer cells that have the capacity for self-renewal, infinite proliferation and tumorigenesis. Collectively, CD13, CD24, CD44, CD90, CD133 and EpCAM are well-accepted stemness markers used to distinguish CSCs from noncancer stem cells. Historically, the manifest characteristic of CSCs confers high chemoresistance to numerous malignancies. Therapy-induced CSC senescence allows for elevated efficacy of traditional agents and prolonged survival rates. Currently, compelling lines of evidence have confirmed the importance of liver cancer stem cells (LCSCs) in mediating the refractoriness of HCC. A brief description of the characteristics of LCSCs and their correlations with ncRNA-related resistance is introduced as follows.

### Critical regulatory elements of LCSCs

Originally, LCSCs were derived from transformed liver progenitor cells, which differentiate into liver parenchymal cells, including hepatocytes and cholangiocytes. Mechanistically, Nanog, Oct4, SALL4 and Sox2 are the dominant genes modulating the stemness of LCSCs. For example, as an essential downstream effector of CD24-associated hepatocarcinogenesis, Nanog can maintain the pluripotency of LCSCs dependent on the STAT3 signaling pathway [19]. Consistently, in hepatic stem cell-like HCC cells, the upregulated transcription level of SALL4 is characterized by the activation of CD44 and EpCAM with a highly carcinogenic and metastatic nature, probably via the Wnt/β-catenin pathway [20]. In particular, the transcription factors Sox2 and Oct4 work in a synergistic network to maintain the self-renewal and stemness of pluripotent stem cells [21]. Recently, the TME has been reported to play an increasing role in preserving stem cell traits, which comprise a complicated system of cells other than cancer cells. Hypoxia is a representative feature of the TME, and hypoxia increases the transcription of ubiquitin-specific protease 22 (USP22) by activating hypoxia-inducible factor-1α (HIF-1α), a key component in stemness maintenance, thereby causing HIF-1α/USP22 positive feedback to repress TP53 expression and stabilize cancer stemness in HCC [22]. Moreover, an increase in stem cell-like properties is also induced by several cytokines, and the inflammatory factors IL6, IL-8, MCP-1, TGF-β, CCL2 and CXCL11 are positively correlated with LCSC markers [23–25]. Li et al. further demonstrated that IL6 plays an important role in the conversion of non-LCSCs to LCSCs via STAT3 signaling, which contributes to chemoresistance in HCC [26]. Similarly, CXCL11, a chemokine overexpressed in HCC, can facilitate cancer stemness by stimulating downstream CXCR3/ERK1/2 signaling [25]. Other components, such as immune cells, cancer-associated fibroblasts (CAFs) and the extracellular matrix, are also closely linked with stemness traits [27]. Ultimately, dysregulated changes in DNA methylation are implicated in liver cancer stemness. Noticeable upregulation of YTHDF2-dependent m6A methylation in the OCT4 mRNA 5′-UTR has been reported in Hep3B and Huh7 cells to support CSC properties [28]. Taken together, epigenetic modifications of stemness-mediated genes, dysregulated levels of relevant signaling pathways and alterations of TME-associated elements are considerable contributors to LCSCs.

### Crosstalk between ncRNAs and LCSC-mediated chemoresistance in HCC

**Fig. 1:**
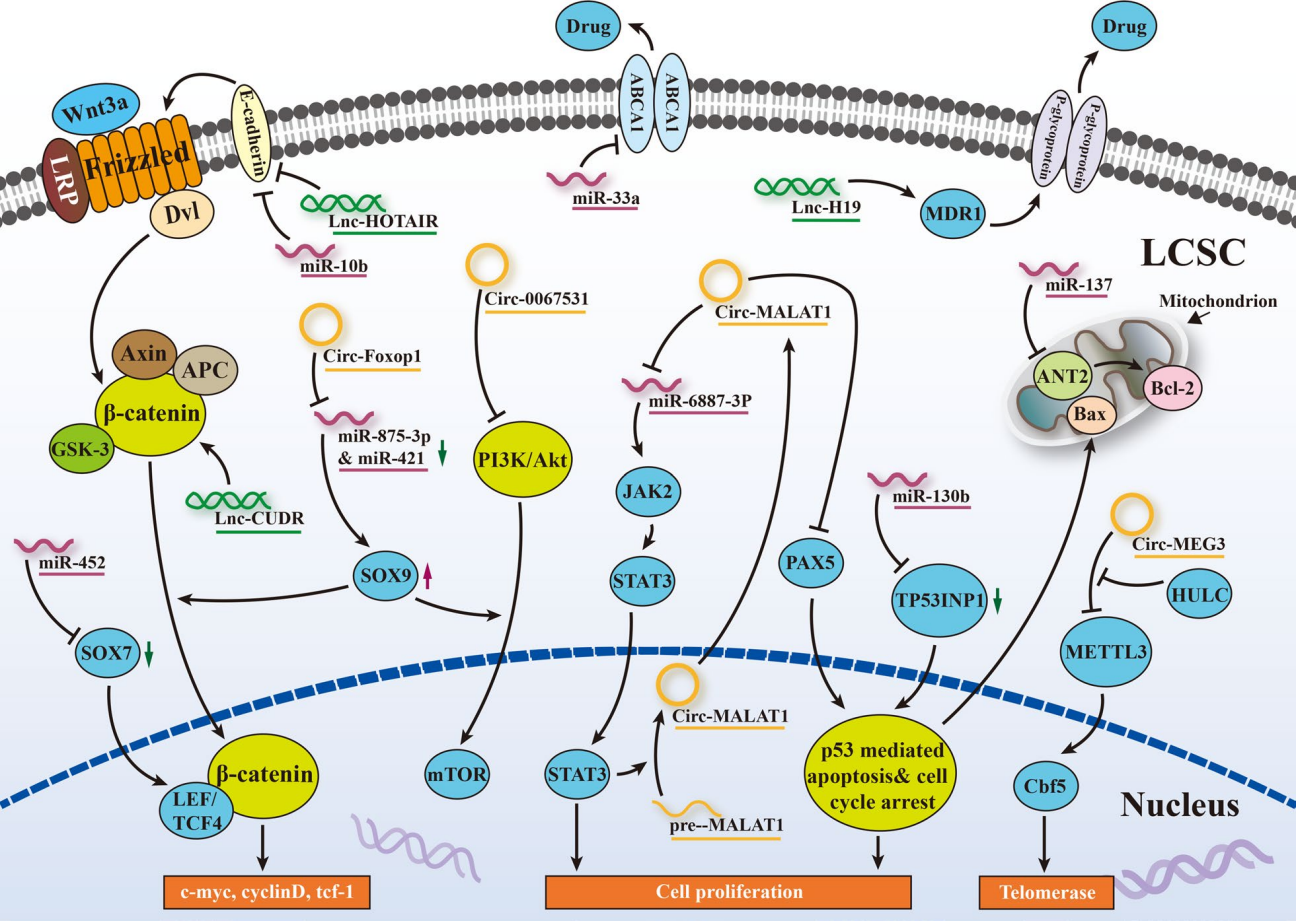
Aberrant expression profiling of ncRNAs enhance stemness maintenance and confer chemoresistance to LCSCs. Aberrant ncRNAs expression could influence the stemness and resistance of LCSCs probably via Wnt/β-catenin signaling, PI3K/Akt signaling, cell cycle arrest, cell proliferation and apoptosis

Plenty of research has been conducted exploring the role of ncRNAs in LCSC-induced chemoresistance. First, data from a range of investigations have suggested that liver cancer cells with CSCs are resistant to common therapies through several regulatory mechanisms, which consist of activated prosurvival signaling pathways, repressed apoptotic pathways, the release of TME-related factors, and enhanced drug efflux and metabolism [26, 29, 30]. Shortly thereafter, as another method of epigenetic modification, aberrant expression profiling of ncRNAs showed that ncRNAs can enhance stemness maintenance and confer chemoresistance to LCSCs. As illustrated in Fig. 1, ncRNAs can be classified into oncogenes or tumor suppressors depending on where they are expressed and what they regulate. MiR-33a is downregulated in Lgr5^+^ HCC-CSCs, and restoring its expression with ectopic mimics could sensitize HCC to doxorubicin by directly targeting the drug efflux-related protein ABCA1 [31]. In the HCC Huh7 cell line, miR-137 functions as a tumor suppressor to inhibit the formation of LSCS traits and reverse sorafenib resistance by degrading adenine nucleotide translocator 2 (ANT2) [32]. Regarding tumor promotors, miR-452 is responsible for stem-like characteristics and chemoresistance by inhibiting SOX7 via the Wnt/β-catenin signaling pathway in HCC [33]. Guan et al. [34] found that miR-130b overexpression not only facilitates CD133^+^ liver-initial cell growth but also increases resistance to a certain degree by silencing TP53INP1. Meanwhile, lncRNA-H19 is enriched in CD90^+^ liver cancer cells, and antisense H19 oligonucleotide transfection induces a remarkable increase in the ratio of MDR1 promoter methylation, which gives rise to doxorubicin accumulation [35, 36]. LncRNA-HOTAIR is of great significance in the malignant transformation from normal liver stem cells to LCSCs via miR-10b-induced EMT activation, which is another factor in drug resistance [37]. Simultaneously, lncRNA-CUDR is positively involved in the malignant transformation of LCSCs through the downstream cascade of HULC/β-catenin signaling [38], which sheds light on the reversal of chemoresistance in HCC. As a newly identified class of ncRNAs, circRNAs with covalently closed single-stranded RNA loop structures play a potential role in the regulation of CSCs. A microarray assay was performed to determine the circRNA profile in the SK‑Hep‑1 cell line, in which hsa_circ_0067531 was significantly decreased in CD90^+^ cells and restrained the sphere-forming ability of LCSCs via the PI3K/Akt pathway [39]. Moreover, circMEG3 inversely correlates with the expression of Cbf5 and shortens the telomere lifespan of LCSCs through METTL3 in a HULC-dependent manner [40]. In particular, as the most generalizable interrelation of the circRNA-miRNA axis, circ-FOXP1 is a driver for the carcinogenesis and progression of LCSCs that concurrently sponges miR-421 and miR-875-3p, leading to an increased level of the target gene SOX9 [41]. Likewise, circ-MALAT1 can act as both a miRNA sponge and an mRNA brake to stabilize the stemness of LCSCs, including miR-6887-3p and PAX5 mRNA [42]. Overall, current and ongoing studies have underscored the significance of ncRNAs in LCSC-induced chemoresistance and provided prospects for HCC therapy.

## Significance of ncRNAs in EMT-related chemoresistance of HCC

### Molecular bases of EMT-related chemoresistance in HCC

Epithelial-mesenchymal transition (EMT), as the name implies, represents a morphogenetic process whereby epithelial cells shed cell polarity and adhesion and acquire properties of migration and invasiveness to become mesenchymal cells. Abnormal EMT triggered by cytokines, growth factors or the TME is characterized by detachment of cell contact, changes in the cytoskeletal network and enhanced proteolytic activity, consequently leading to tumorigenesis and drug resistance. In terms of HCC, as a submember of the TGF-β family, BMP4 was reported by Shen et al. to accelerate mesenchymal marker-induced oxaliplatin resistance via MEK/ERK/ELK1 signaling activation [43]. As the critical downstream element of hepatocyte growth factor (HGF), MET is a substantial contributor to EMT-associated resistance in HCC mainly through autophagy and the canonical PI3K/Akt axis, whether in vivo or in vitro [44, 45]. More importantly, as a ubiquitous feature of solid tumors, hypoxia exhibits a positive correlation with EMT-induced cisplatin refractoriness via the PI3K/Akt/HIF-1α pathway, such that suppression of the aforementioned axis will undoubtedly enhance the therapeutic outcomes of hypoxic chemotherapy in HCC [46]. Intriguingly, a potential link between EMT and CSCs has been recently described: coexpression of Oct4/Nanog in LCSCs accelerates EMT via upregulation of the STAT3/Snail pathway [47], which reveals another regulatory base of EMT-involved resistance. As described above, EMT is closely related to HCC chemoresistance through different molecular machinery.

### Interplay between ncRNAs and EMT-associated resistance in HCC

Over a dozen observations have revealed that ncRNA-mediated EMT may also regulate therapeutic refractoriness, thus paving a new avenue for the identification of modulated mechanisms and suggesting a latent paradigm for HCC treatment. The profiles of ncRNA expression are tissue- and cell type dependent-, as shown in Table 1. Concerning miRNAs, miR-212-3p is viewed as a tumor inhibitor in the Huh7 cell line, and its overexpression effectively counteracts paclitaxel resistance by targeting ZEB2, a key factor in EMT [48]. Analogously, miR-106a has been reported to act as a link between PDGF-D and Twist1, and restoration of miR-106a levels reverses EMT-mediated gemcitabine resistance in HCC [49]. In line with these findings, Wang et al. [50] emphasized the ability of miR-9 to enhance the sensitivity of HCC to cisplatin by regulating EIF5A2-related EMT. In contrast, a new cluster of miR-216a/217 was found to be positively associated with the EMT phenotype and sorafenib resistance through the TGF-β and PI3K/Akt signaling pathways by targeting PTEN and SMAD7, respectively [51]. Subsequently, with respect to lncRNAs, a previous study posited the positive effect of lincRNA-ROR on doxorubicin resistance by interacting with the mesenchymal cell marker, Twist1 [52]. In addition, lncRNA-H19 can either directly function by targeting the MDR1 gene [36] or indirectly sponge miR-675 [53] to aggravate EMT-mediated chemoresistance in HCC. Strikingly, lncRNA-POIR, a competing endogenous RNA (ceRNA), was determined to function as a molecular sponge to silence miR-182-5p [54], which was identified as a negative mediator of EMT-associated sorafenib insensitivity. Similarly, EMT-mediated sorafenib resistance may also be induced by lncRNA-SNHG3 via modulation of the miR-128/CD151 pathway in HCC [55]. Nevertheless, the importance of anti-EMT lncRNAs in the chemoresistance of HCC remains to be further explored. Current evidence shows that the antitumor lncRNA-AOC4P and lincRNA-p21 suppress EMT-induced HCC metastasis via degradation of vimentin [56] and inhibition of Notch signaling [57], suggesting a reasonable clue for the potential therapeutic target of HCC chemoresistance. Ultimately, the mechanisms underlying circRNAs and EMT-related resistance have been clarified in several studies [58, 59]. Specifically, circ_0003998 is elevated in doxorubicin-resistant HCC cells and serves as a ceRNA to downregulate miR-218-5p, which is negatively associated with the content of the EMT marker EIF5A2. Consistently, circFoxo3 is overexpressed in HCC samples and promotes adriamycin resistance via the miR-199a-5p/ABCC1 axis, contributing to EMT progression. Altogether, we summarize the basic intercorrelation between ncRNAs and EMT-involved resistance, and further efforts are required to probe deeper mechanisms and address refractoriness in HCC.

**Table 1:** Interplay between ncRNAs and EMT-associated resistance in HCC

ncRNAs		Change	Element affected	Consequence	References
MiRNAs	miR-212-3p	Down-regulation	Paclitaxel	Reduced ZEB2 level	[48]
	miR-106a	Down-regulation	Gemcitabine	Reduced PDGF-D/Twist1 pathway	[49]
	miR-9	Down-regulation	Cisplatin	Reduced EIF5A2-mediated EMT	[50]
	miR-216a/217	Up-regulation	Sorafenib	Induced SMAD7/TGF-β & PTEN/PI3K/Akt signaling	[51]
LncRNAs	Linc-ROR	Up-regulation	Doxorubicin	Induced Twist1 level	[52]
	Lnc-H19	Up-regulation	Sorafenib	Induced EMT via sponging miR-675	[53]
	Lnc-POIR	Up-regulation	Sorafenib	Induced EMT via sponging miR-182-5p	[54]
	Lnc-SNHG3	Up-regulation	Sorafenib	Induced EMT via miR-128/CD151 pathway	[55]
	Lnc-AOC4P	Down-regulation	EMT	Reduced vimentin level	[56]
	Linc-p21	Down-regulation	EMT and lung metastasis	Inhibited Notch signaling	[57]
CircRNAs	Circ_0003998	Up-regulation	Doxorubicin	Induced EIF5A2-mediated EMT via sponging miR-218-5p	[58]
	CircFoxo3	Up-regulation	Adriamycin	Induced ABCCA1-mediated EMT via sponging miR-199a-5p	[59]

## Participation of ncRNAs in autophagy-involved resistance of HCC

### The molecular machinery of autophagy-mediated chemoresistance in HCC

Autophagy is characterized as an evolutionarily conserved procedure in which damaged proteins, aberrant organelles and excessive cytoplasmic components are degraded via lysosomal or vacuole-dependent pathways. On the basis of delivery modes, there are three main forms of autophagy: microautophagy, macroautophagy and chaperone-mediated autophagy. As the most studied form, macroautophagy usually occurs constitutively at a basal level and can be further induced by various metabolic stresses. For brevity, macroautophagy henceforth referred to as autophagy, involves the formation of autophagosomes in the cytoplasm, fusion with lysosomes or vacuoles and degradation of autolysosomes by proteases or nucleases, which is regulated via autophagic essential genes (ATGs, Beclin1, ULK1, LC3 complex, etc.) and signaling pathways to sustain the microenvironment. More importantly, dysfunctional autophagy may be a necessary regulator not only in the development of different cancers but also in determining the efficacy of antitumor chemotherapy. For HCC, on the one hand, basal autophagy is reported to remove damaged mitochondria and mutated cells to maintain genomic stability in the dysplastic phase of hepatocytes; on the other hand, dysregulated autophagy continues to sustain HCC cell survival when a solid tumor is established [60]. Considering the paradoxical role of autophagy in HCC, therapy choice varies depending on the density of hepatocytes. Currently, with a growing number of studies performed on the relationship between autophagy and chemoresistance, we coincidentally determined the positive effect of autophagy on preventing HCC cells from traditional therapies through multiple mechanisms. Accordingly, as a representative feature of the TME, oxidative stress may also act as a driver of autophagy-induced chemoresistance via the Egr-1/LC3 [61] and FOXO3a/Beclin-1 [62] axes. As another component of the TME, tumor-associated macrophages (TAMs) induce autophagy, and inhibition of ATG5 expression and LC3 conversion enhances the efficacy of oxaliplatin in TACE [63]. Interestingly, the stemness-related marker CD13 was reported by Wang et al. [12] to promote HCC resistance via the P38/Hsp27/CREB/ATG7 pathway, which uncovers the intercross between autophagy and LCSCs. In general, induced autophagy contributes to chemoresistance to a certain degree through diverse machinery.

### Regulatory mechanism of ncRNAs in autophagy-mediated HCC resistance

**Fig. 2:**
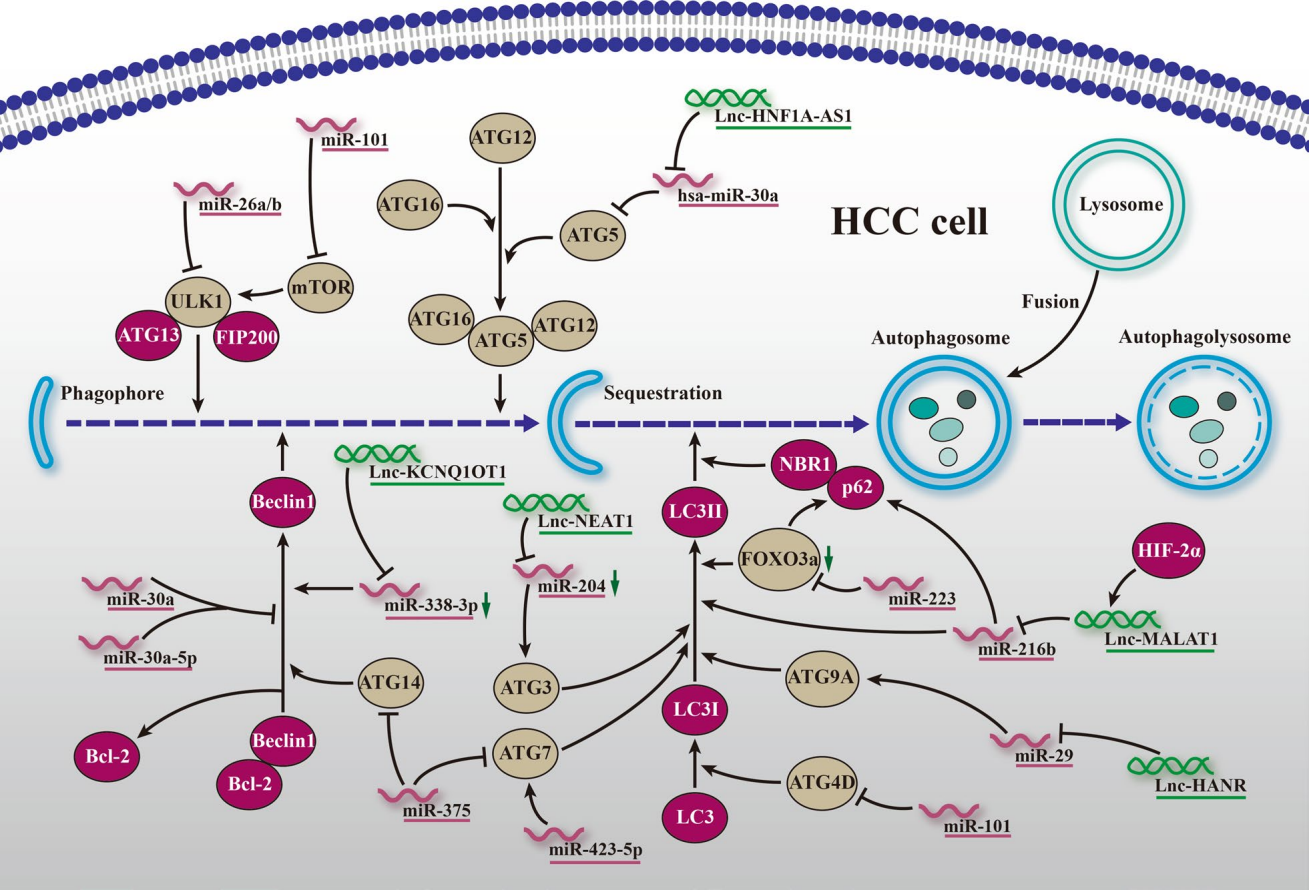
Regulatory mechanism of ncRNAs in autophagy-mediated HCC resistance. ncRNAs may regulate the refractoriness with regard to the autophagy related genes, including ULK1, Beclin1, ATG3, ATG4D, ATG5, ATG7, ATH9A, ATG14, LC3 and p62

As described previously, induced autophagy exerts a protective effect from current chemotherapeutic agents in established HCC, whether in cells or samples. During the past few decades, growing enthusiasm has been geared toward investigating the characteristics and activities of ncRNAs in autophagy. According to the density of ncRNAs, they are classified as either promoting or inhibiting autophagy and are depicted in Fig. 2. Li et al. [64] found that the miR-26a/b level was downregulated and accompanied by induced autophagy in doxorubicin-resistant HCC cells. In turn, restoration of ULK1 expression inhibited ULK1-initiated autophagy and promoted cell apoptosis by doxorubicin. In particular, the tumor suppressor miR-375 plays a significant role in autophagy inhibition in that it can inhibit the formation of autophagosome-fused lysosomes and prolong the residence duration of sorafenib [65]. Mechanistically, miR-375 may also promote the sensitivity of HCC cells to sorafenib in a manner dependent on ATG14 regulation [66]. Moreover, insight into the role of miR-233 in doxorubicin-initiated autophagy uncovered the resistance mechanism by which it suppresses FOXO3-mediated autophagy in HCC [67]. Moreover, miR-101 is reported to block tumor progression in vivo [68] and prohibit the formation of autophagosomes by reducing ATG4D expression and mTOR signaling in vitro [69], showing that the presence of miR-101 attenuates autophagy and chemoresistance in HCC. Consistently, hsa-miR-30a and its subtype hsa-miR-30a-5p are inversely related to autophagy via the ATG5 and Beclin1 axes, thereby countering anoikis [70] and drug resistance [71] in HCC. Conversely, pro-autophagic miR-423-5p is upregulated in sorafenib-treated HCC cells and samples, and ectopic mimics facilitate autophagosome and vacuole formation, implying its potential to predict the response of patients to sorafenib [72]. Subsequently, lncRNAs have been shown to mediate the interplay between autophagy and resistance. Strikingly, the existing lncRNAs are all pro-autophagic in HCC. By comparison with normal SK-HEP-1 cells, Huang et al. found that the content of lncRNA-KCNQ1OT1 was statistically higher in cisplatin-resistant cells. The authors further determined that knockdown of lncRNA-KCNQ1OT1 overcomes autophagy-mediated resistance by leveraging the miR-338-3p/Beclin1 axis [73]. Upstream of hsa-miR-30a-5p, lncRNA-HNF1A-AS1 functions as an autophagy promoter to induce hepatocarcinogenesis via ATG5 regulation [74], which may be a promising therapeutic target in the reversal of drug resistance. Similarly, lncRNA-NEAT1 and lncRNA-HANR competitively bind with corresponding miR-204 and miR-29b, thus stimulating autophagy-initiated resistance via ATG3 [75] and ATG9A [76], respectively. Finally, lncRNA-MALAT1 induced by HIF-2α is upregulated in 5-FU-resistant Bel-7402 cells, and transfection of MALAT1 siRNA or miR-216b mimics leads to reduced LC3-II levels and increased p62 accumulation, implying the significance of the HIF-2α/MALAT1/miR-216b axis in autophagy-related chemoresistance of HCC [77]. However, to the best of our knowledge, there exists no literature on the role of circRNAs in autophagy-related resistance, which provides possibilities for subsequent studies. Aside from the aforementioned molecular machinery of ncRNAs in nonselective autophagy-mediated resistance of HCC, it is urgent to explore detailed mechanisms of ncRNA-mediated refractoriness underlying selective autophagy, such as mitophagy, reticulophagy, pexophagy and lipophagy.

## ncRNA-containing extracellular vesicles and chemoresistance in HCC

Extracellular vesicles (EVs) are defined as a heterogeneous category of membrane-bound vesicles that encompass lipids, proteins, mRNAs, miRNAs, lncRNAs, circRNAs and transcription factors and are classified as exosomes (diameter from 40 to 100 nm), microvesicles (MVs, diameter from 100 nm to 1 μm) and apoptotic bodies (diameter from 1 to 4 μm). EVs are generated and released upon cell activation or apoptosis with various components depending on the original cellular and the stimulus for their formation. As the main form of biological communication, EV delivery of ncRNAs in liver cancer has been thoroughly reviewed. Herein, as the third molecular regulatory tool, we summarize the functions of these exosomes and MV-containing biomolecules in the regulation of progression and therapeutic efficacy in HCC, and the potential machinery is clarified as follows in Table 2.

Specifically, the expression of exosomal ncRNAs is significantly associated with the development and chemoresistance of HCC. Accordingly, serum exosomes from HCC patients show a notably lower level of miR-9-3p. By bioinformatics analysis, the authors identified the target gene HBGF-5, through which miR-9-3p reduced cell viability and ERK1/2 signaling [78]. Meanwhile, in HCV-related HCC, activated mast cells can transfer exosomal miR-490 to cancer cells and restrain the EGFR/Akt/ERK1/2 pathway to shuttle HCC metastasis [79]. Furthermore, Dou et al. [80] demonstrated the loss of exosomal miR-320a in activated CAFs and the promotion of malignant phenotype transition via the ERK1/2 axis, establishing additional insight into the exploration of exosomes and the TME. Conspicuously, miR-122 is closely related to a superior prognosis of HCC, and exosome-mediated miR-122 transfer to HepG2 cells increases sensitivity to sorafenib by targeting CCNG1, ADAM10 and IGF1R [81]. Interestingly, as an important host risk factor for HCC, HCV RNA accumulation is promoted by miR-122 in hepatitis C patients. Decreased intrahepatic contents of miR-122 were detected in HCC patients, indicating a potential role of miR-122 during the development of HCC. Reportedly, a clinical trial demonstrated that the miR-122 antagonist Miravirsen was developed for the management of HCV, and phase I and II studies displayed its dose-dependent pharmacology with mild side effects [82], which is of great importance for the evaluation of anti-HCC therapy. Regarding protumor exosomal miRNAs, a recent study reported that exosome-containing miR-1247-3p from HCC cells could facilitate remote lung metastasis and conversion of CAFs through β1-integrin-NFκB signaling [83]. Similarly, a cluster of miR-21 and miR-10b secreted from exosomes was induced in the acidic milieu and promoted HCC cell proliferation and metastasis [84]. In addition, it is hypothesized that circulating miR-32-5p induces MDR in HCC via exosomes through the PTEN/PI3K/Akt axis [85], which is related to angiogenesis, proliferation, EMT and autophagy.

On the other hand, exosomal lncRNAs possess unique expression profiles characterizing the progression and therapeutic resistance of HCC. To be concrete, along with exosomal miR-21, lncRNA-ATB secreted from exosomes is positively related to the TNM stage and unfavorable prognosis of patients, which provides considerable value in the therapeutic prediction of HCC [86]. In parallel, serum levels of lncRNA-ENSG00000258332.1 and LincRNA-00635 [87] from exosomes are remarkably higher in HCC patients, which correlates with lymph node metastasis, portal vein tumor emboli, TNM stage and OS. Tash et al. [88] found that exosomal lncRNA-RP11-513I15.6 enhances the level of aggressiveness and metastasis in HCC via its competitive endogenous exosomal miR-1262/RAB11A network. Additionally, a previous study reported that CD90^+^ liver cancer cell-derived exosomes could facilitate angiogenesis in the TME, and lncRNA profiles revealed that lncRNA-H19 is enriched in exosomes and acts as a possible mediator of angiogenic effects [35]. For circRNAs, an increased level of exosomal circ-UHRF1 indicates unfavorable outcomes in patients with HCC. Moreover, Ke et al. [89] demonstrated the delivery of circ-UHRF1 via exosomes to NK cells and the induction of NK-cell exhaustion by sponging miR-499c-5p, leading to refractoriness to anti-PD1 immunotherapy.

Regarding MVs, several studies have elucidated that MV-mediated transfer of lncRNAs represents a distinct mechanism to modulate the TME, tumor proliferation and chemoresistance in HCC. Specifically, MV-containing lncRNA-TUC339 has been implicated in hepatocarcinogenesis via intercellular signaling mediators [90], which makes such extracellular lncRNA a promising marker for HCC. In addition, Patel et al. [91] reported the enrichment of lincRNA-ROR within MVs derived from HCC cells. Incubation of MV-containing lincRNA-ROR and sorafenib suppresses chemotherapy-induced cell apoptosis by interacting with TGF-β, which is also a critical characteristic of LCSCs.

In this chapter, we systematically summarize the implications of EV-containing ncRNAs in the progression and drug sensitivity of HCC. Nevertheless, further efforts are warranted to elucidate the molecular bases underlying the importance of circRNAs, especially EV-related circRNAs.

**Table 2:** ncRNA-containing extracellular vesicles and chemoresistance in HCC

ncRNAs		Change	Element affected	Consequence		References
Exosomal-miRNAs	miR-9-3p	Down-regulation	HBGF-5		Reduced HCC proliferation via ERK1/2 level	[78]
	miR-490	Down-regulation	ERK1/2		Reduced HCC metastasis via EGFR/Akt axis	[79]
	miR-320a	Down-regulation	EMT, CDK2, MMP2		Reduced PBX3 mediated ERK1/2 axis	[80]
	miR-122	Down-regulation	Sorafenib		Induced drug sensitivity via CCNG1, ADAM10 and IGF1R	[81]
	miR-1247-3p	Up-regulation	CAFs and lung metastasis		Induced B4GALT3/β1-integrin-NFκB axis	[83]
	miR-21 and miR-10b	Up-regulation	Angiogenesis		Induced HCC proliferation and metastasis via HIF-1α and HIF-2α	[84]
	miR-32-5p	Up-regulation	Sorafenib, Oxaliplatin, 5-FU, Gemcitabine		Induced MDR via PTEN/PI3K/Akt axis	[85]
Exosomal-lncRNAs	Lnc-ATB	Up-regulation	Sorafenib		Elevated miR-21 and poor TMN stage	[86]
	Linc-00635 and Lnc-ENSG00000258332.1	Up-regulation	lymph node metastasis, portal vein tumor emboli		Unfavorable TMN stage and OS	[87]
	Lnc-RP11-513I15.6	Up-regulation	RAB11A		Induced HCC progression via sponging miR-1262	[88]
	Lnc-H19	Up-regulation	Doxorubicin		Induced CD90^+^ HCC angiogenesis	[35]
Exosomal-circRNAs	Circ-UHRF1	Up-regulation	ICIs		Induced NKs exhaustion and PD-1 level via sponging miR-499c-5p	[89]
MVs-lncRNAs	Lnc-TUC339	Up-regulation	HCC cell behavior		Reduced cell adhesion and induced HCC growth	[90]
	Linc-ROR	Up-regulation	Sorafenib		Reduced apoptosis via TGF-β signaling	[91]

## Intercorrelation between ncRNAs and immune evasion in HCC

Recently, immunotherapy has become a research hotspot in terms of investigations into immunotherapeutic agents that confer a notable prognostic benefit by altering the immune microenvironment to develop an effective response against several malignancies. Indeed, its combination with traditional agents may change the treatment landscape. However, as the main immune organ, the liver plays an overarching role in host defense and is characterized by a strong immunosuppressive microenvironment and high immune evasion, which creates a vital impediment to an efficacious immune response. Considering the density of immune cells, chemokines and cytokines, the immune microenvironment may elicit dual activating and suppressive roles in tumor progression or tumor eradication. Specifically, liver sinusoidal endothelial cells (LSECs), Kupffer cells (KCs), regulatory T cells (Tregs) [92], dendritic cells (DCs), myeloid-derived suppressor cells (MDSCs) [93], natural killer cells (NKs) [94], immune checkpoints PD-1/PD-L1 [95], CTLA4 [96], lymphocyte activation gene 3 (LAG-3), glypican-3 (GPC-3), galectin 9 [97], immune-related cytokines [98] IL1, TNF-α, IFN-γ, IL-4, IL-5, IL-8 and IL10 are significant factors engaging in the maintenance of immune balance, which provides some hints for HCC immunotherapy. In this framework, a thorough understanding of the immune microenvironment and ncRNA-mediated immune evasion will assist to counterbalance immunosuppression in HCC treatment.

### Biofunction of ncRNAs in HCC immunotherapy

**Fig. 3:**
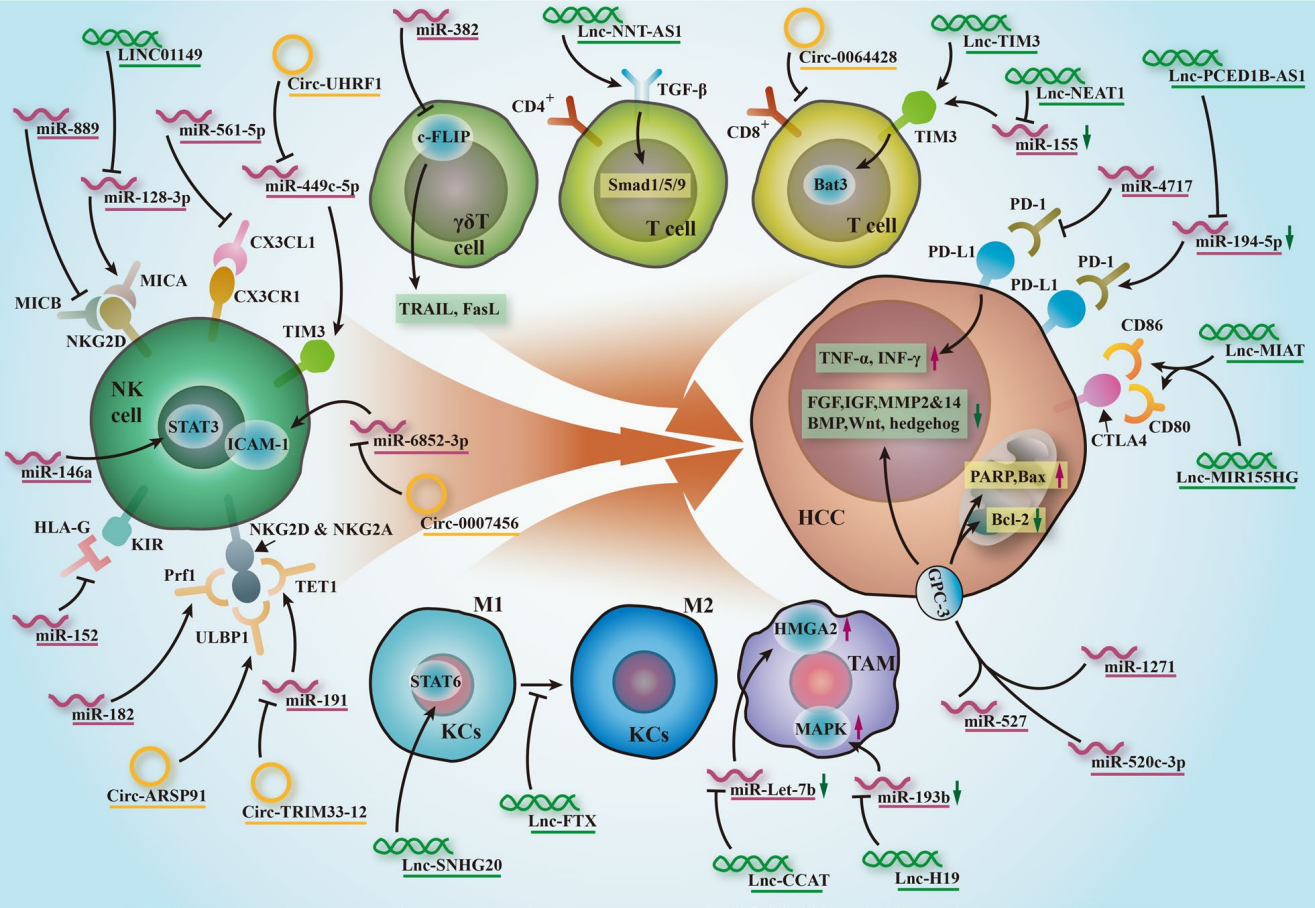
Biofunction of ncRNAs in HCC immunotherapy. ncRNAs are involved in the immune evasion that compromises NKs, KCs, TAMs, CD4 + T, CD8 + T, γδ T cells, GPC-3, PD-1, PD-L1, CTLA4, chemokines and cytokines

Currently, numerous studies have highlighted the immunomodulatory effects of ncRNAs that are involved in immune cells, immune checkpoint molecules, chemokines, cytokines, immunosuppressive signaling pathways, etc. According to Fig. 3, preclinical studies have provided intuitive evidence that miRNAs participate in HCC immune escape from NK-cell activity. As a novel targeting miRNA of MICB, miR-889 is overexpressed in HCC cell lines and confers resistance to NK-mediated cytotoxicity [99], and a specific histone deacetylase inhibitor can enhance the susceptibility of HCC to NK-based immune strategies. Similar to miR-889, miR-561-5p is overexpressed in HCC tissue with high metastatic potential and accelerates pulmonary metastasis by regulating CX3CR1^+^ NKs via the CX3CL1/STAT3 signaling axis [100]. Comparatively, in HBV and HVC-related HCC, miR-152 is reported to be positively correlated with the degree of NK cytolysis against tumor cells, probably by facilitating the degradation of the immunotolerance molecule HLA-G [101]. Coincidently, Abdelrahman et al. [102] also found that miR-182, an immunoinhibitory miRNA, could counter the imbalance between the activating receptor NKG2D and the inhibitory receptor NKG2A, which ultimately augments NK lytic activity in HCC immunotherapy. As another immune cell, the efficacy of γδ T-cell-based immunotherapy was amplified by the forced overexpression of miR-382 [103], which directly inhibited the antiapoptotic member c-FLIP and triggered caspase-8 mediated apoptosis. In addition, the intercross between miRNAs and peptide GPC-3-related vaccine therapy is described as follows. As an oncofetal glycoprotein, GPC-3 is overexpressed and associated with a poor prognosis in HCC. A microarray test revealed that miR-527 is an upstream effector of GPC-3, and transfection of miR-527 suppressed the invasiveness of HCC [104]. Consistently, by targeting GPC-3, miR-520c-3p can play an inducible role in HCC cell apoptosis [105], therefore inhibiting proliferation and invasion. A recent functional screening also revealed a suppressive effect of miR-1271 on GPC-3 by binding to the 3′-UTR [106], which constitutes a promising avenue of anti-HCC therapy. Thus, the clarified significance of miRNAs in GPC-3 will pave a new way for the improvement of vaccine therapy. In addition, Zhang et al. [107] found a negative relationship between miR-4717 and PD-1 in that HBV-induced release of PD-1 was putatively abolished by miR-4717, which increased the production of TNF-α and IFN-γ and blocked transformation of hepatocarcinogenesis resulting from HBV infection. As a key signaling pathway regulating immune suppression, the STAT3 axis is responsible for the transcription of miR-146a [108]. Importantly, inhibition of miR-146a not only modifies the STAT3-associated cytokine profile but also attenuates HCC-induced NK dysfunction and enhances the antitumor activity of lymphocytes. More impressively, a liposome-based miR-34 mimic, MRX34, has been investigated in phase I clinical trial for the treatment of HCC patients. MiR-34 plays an important tumor-suppressive role by downregulating the immune checkpoint PD-L1 and multiple oncogenes, including CD44, MET, CDK4/6 and NOTCH. Preclinical studies have demonstrated the therapeutic effects of MRX34, which include reduced tumor volume and enhanced survival in orthotopic mouse models of HCC [109]. An update on MRX34 during the ASCO Annual Meeting 2016 clarified its manageable toxicity profile and evident antitumor activity in liver cancer. However, the clinical trial has been at a standstill due to several immune-associated severe adverse effects among the patients, which remains to be further evaluated and investigated in the field of miRNAs-related therapy.

In the context of lncRNAs, lncRNA-FTX [110] upregulates the M1/M2 KC ratio and inhibits the NAFLD conversion to HCC. However, oncogenic lncRNA-SNHG20 promotes the aforementioned progression by inducing M2 polarization of KCs via STAT6 activation [111]. A functional enrichment analysis evaluated the critical role of lncRNA-GIHCG in HCC progression with negative regulators in the infiltration of DCs, NKs, CD4^+^ and CD8^+^ T cells [112]. Moreover, another genome-wide association study showed that LINC01149 competitively sponges miR-128-3p and releases highly soluble MICA, thus inducing NK exhaustion and immune evasion [113]. Moreover, immunosuppressive TAMs are positively correlated with the expression of HMGA2, which is mediated by the lncRNA-CCAT1/miR-Let7b axis [114]. Induced TAMs may also further increase the level of lncRNA-H19 and trigger the downstream miR-193b/MAPK1 pathway to promote HCC aggressiveness [115]. In addition, as a typical immunogenicity marker, TIM3-mediated CD8^+^ T cell activity is determined by lncRNA-Tim3 [116] and lncRNA-NEAT1 [117]. LncRNA-TIM3 exacerbates CD8^+^ T-cell exhaustion to compromise antitumor immunity by binding to TIM3, and repression of lncRNA-NEAT1 amplifies the anticancer effect of CD8^+^ T-cells against HCC via the miR-155/Tim-3 axis. In terms of immune checkpoints, lncRNA-MIAT and lncRNA-MIR155HG are remarkably related to PD-1, PD-L1 and CTLA-4, which are engaged in the immune escape process of HCC [118, 119]. Next, lncRNA-PCED1B-AS1 is highly expressed in HCC cells and exosomal lncRNA-PCED1B-AS1 elevates PD-Ls-related immunosuppression by sponging hsa‑miR‑194‑5p [120]. In regard to cancer vaccines, by epigenetically activating the oncogene GPC-3 through PCAF recruitment [121], lncRNA-GPC3-AS1 was found to enhance HCC cell proliferation and migration. Of note, lncRNA-HOXA-AS2 promotes GPC-3-related proliferation and EMT in HCC via the miR-520c-3p/GPC3 interaction [122]. For ACT therapy, Wang et al. found that lncRNA-NNT-AS1 reduced the level of CD4^+^ TILs via activation of the TGF-β pathway in HCC [123].

Regarding circRNAs, several substantial studies have also provided comparable findings. CircARSP91 can enhance innate immune surveillance by strengthening NK cytotoxicity by upgrading ULBP1 in HCC immunity [124]. Likewise, circ_0007456 further assists in avoiding immune escape by magnifying NK-mediated immunomodulation through the miR-6852-3p/ICAM-1 axis [125]. As indicated before, Ke et al. [89] found an immunosuppressive effect of circ-UHRF1 in inducing NK exhaustion and inhibiting IFN-γ and TNF-α secretion, thus causing anti-PD-1 resistance in HCC via miR-449c-5p/TIM3, which reveals an interplay between exosomes and immune evasion through circRNAs. In addition, circTRIM33-12 exerts its antitumor role in activating NKG2D in γδ T cells, CD8^+^ T cells and NKs by guaranteeing TET1 from miR-191 [126]. In particular, circ_0064428 is reported to be inversely related to CD8^+^ TILs [127], which potentially reflects the sensitivity and specificity of ACT-based immunotherapy in HCC. Overall, data from initial immunotherapy trials indicate that the specificity of ncRNAs is of paramount importance.

## Biological nexus between ncRNA and ferroptosis in HCC

Ferroptosis, a newly coined form of regulated cell death, is defined as an iron-dependent process that is characterized by the accumulation of lipid peroxides and reactive oxygen species (ROS) under oxidative conditions. As a nonapoptotic cell death process, ferroptosis shares different molecular mechanisms with apoptosis, which provides a new perspective for cancer therapeutics. Historically, traditional anticancer drugs have promoted cell death, likely via caspase-dependent apoptosis. However, tumoricidal efficacy is far from satisfactory due to apoptosis resistance, which occurs frequently in various cancer cell types. Currently, with the fast-growing investigations of ferroptosis in cancer, it is gradually recognized as an adaptive feature to effectively eliminate malignant cells, which may circumvent the limitations of HCC therapy.

Mechanistically, ferroptosis is triggered by iron accumulation and polyunsaturated fatty acid (PUFA) and cysteine metabolism. Multiple classic pathways and a series of molecules are important causative factors in ferroptosis regulation. Normally, intracellular iron maintains an exquisite balance via iron transport systems. Either decreased iron export or increased iron import will sensitize cancerous cells to oxidative stress. As a vital factor in ferroptosis induction, the labile iron level will be elevated with transferrin-mediated iron import or ferritinophagy which is mediated by NCOA4, a specific autophagy cargo that transfers ferritin to lysosomes for the release of free iron [128]. Cystine and glutamate are important amino acids for ferroptosis induction, both of which need to be exchanged across the cell membrane with the specific transporter system Xc^−^, a disulfide-linked heterodimer that consists of SLC3A2 and SLC7A11 [129]. Under the oxidative conditions of cancer cells, the exchange of cystine with glutamate ranks as the most upstream event in ferroptosis, which could be triggered by the inhibition of system Xc^−^. Reportedly, several genes such as GLS1, GLS2, p53 and BAP1, have been identified to regulate SLC7A11 [130, 131], thus influencing cystine uptake and ferroptosis. As the major intracellular antioxidant, glutathione (GSH) is generated from cysteine, glutamate and glycine under the catalysis of glutathione synthetase (GSS). Ferroptosis can be initiated with the depletion of GSH, which can be achieved either by the inhibition of GSH biosynthesis or by the blockage of cystine acquisition via system Xc^−^. Interestingly, the CSC marker CD44 is reported to stabilize system Xc^−^, suggesting a potential link between CSCs and ferroptosis that contributes to cancer treatment [132]. Subsequently, ferroptosis is accompanied by iron-dependent ROS accumulation and PUFA depletion. PUFAs are more susceptible to lipid peroxidation, which results in a variety of products that drive cells to ferroptotic death [133]. As a dominant downstream molecule, GPX4 combats lipid peroxides by reducing GSH to leave oxidized GSH as a byproduct, and silencing of GPX4 facilitates ferroptosis [134]. Incidentally, the Ras/Raf/MEK and p62-Keap1-Nrf2 pathways are reported to be decisive targets for ferroptosis in HCC [135, 136]. In addition, several posttranslational modifications of the mentioned genes also play considerable roles in ferroptosis, including methylation, acetylation, phosphorylation and ubiquitination [137]. Therefore, based on the above signatures, the following tactics could be established to promote ferroptosis in HCC.

**Fig. 4:**
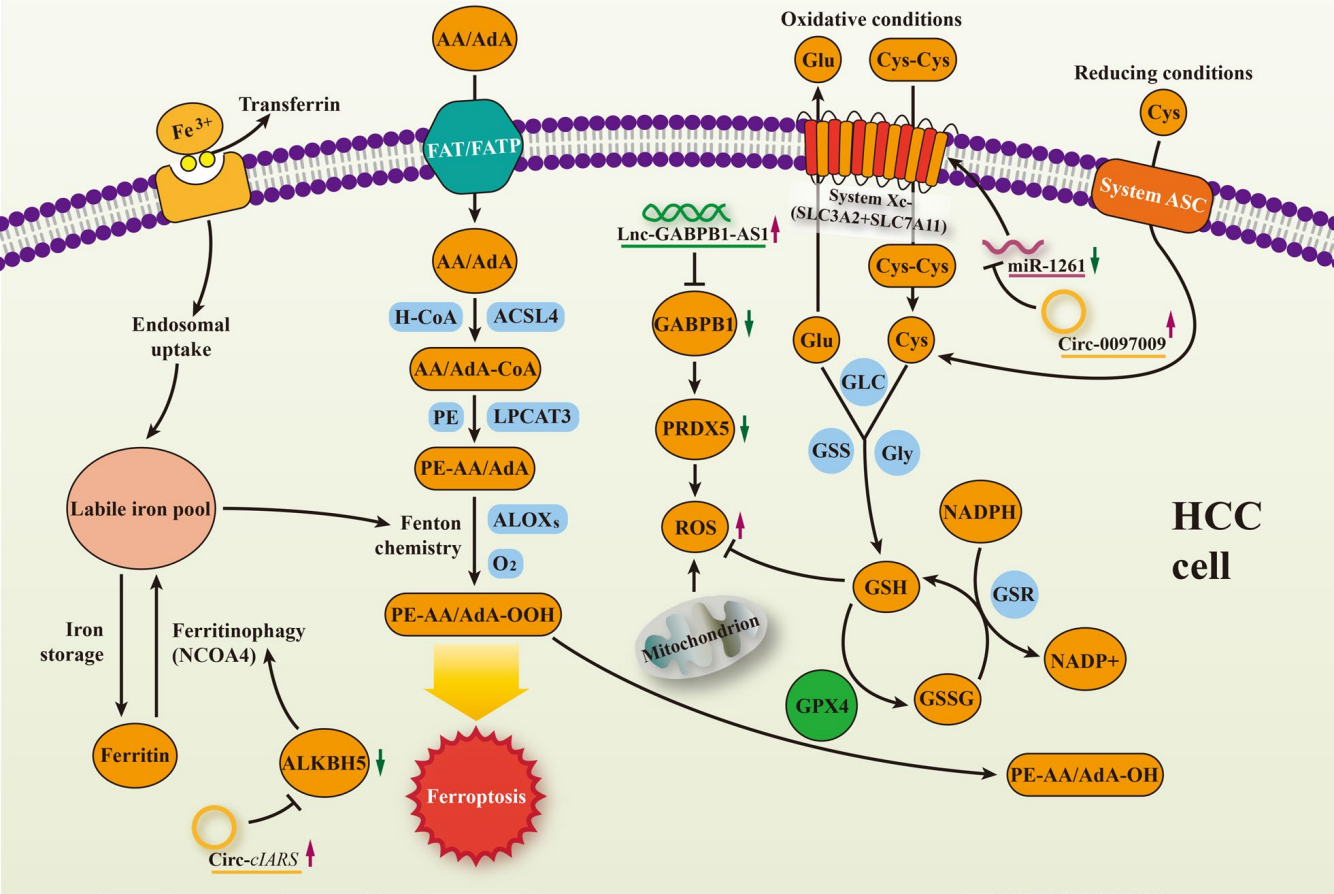
Biological interrelation between ncRNAs and ferroptosis in HCC. Phosphatidylethanolamines (PEs) containing arachidonic acid (AA) and adrenic acid (AdA) are domination substrates that are involved in oxidation and ferroptosis. With the help of fatty acid transport protein (FATP) and fatty acid translocase (FAT), AA and AdA are absorbed via cellular membrane. long-chain acyl-CoA synthetase member 4 (ACSL4) and lysophosphatidylcholine acyltransferase 3 (LPCAT3) are responsible for the esterification and incorporation of PUFAs. Iron-dependent arachidonate lipoxygenases (ALOXs) and Fenton chemistry participate in the synthesis of phospholipid hydroperoxides (PE-AA-OOH/PE-AdA-OOH). GPX4 combats with lipid peroxidation through degrading toxic PE-AA-OOH/PE-AdA-OOH to nontoxic phospholipid alcohols (PE-AA-OH/PE-AdA-OH)

In HCC, several studies have pinpointed the significance of ncRNAs in ferroptosis regulation. Accordingly, as an activation subunit of Nrf2, GABPB1 is downregulated by lncRNA GABPB1-AS1 at the translational level, leading to the suppression of PRDX5-mediated antioxidant capacity and elevated ferroptosis in HCC [138]. Liu et al. [139] demonstrated a ferritinophagy-related circRNA in which *cIARS* positively regulates sorafenib-mediated ferroptosis by preventing ALKBH5-induced autophagy inhibition. A microarray study revealed that circ0097009 is involved in the regulation of SLC7A11, a key mediator in ferroptosis, by binding to miR-1261 in HCC [140]. Based on the current research on the interrelation between ncRNAs and ferroptosis depicted in Fig. 4, we boldly speculate that ncRNAs could regulate HCC progression by mediating ferroptosis, thereby proposing a new molecular mechanism for chemoresistance reversal.

## Conclusions

In conclusion, we have summarized a substantial amount of available information generated by the current broadly active investigations in the field of ncRNA-related chemoresistance in HCC. Remarkably, a variety of these pathological characteristics are intertwined and are involved in the lack of an effective response to common therapies in HCC. This review aims to improve the understanding of the dynamic and complex processes of drug refractoriness, which is indispensable to define novel markers with considerable sensitivity and selectivity that will be useful to precisely predict prognosis. In this regard, the detection of ncRNAs in the biomodulation of the above features, in both fundamental and applied aspects, will prove essential in motivating future innovations for HCC treatment.

## Data Availability

Not applicable.

## Abbreviations

ncRNAs: Noncoding RNAs
HCC: Hepatocellular carcinoma
CSCs: Cancer stem cells
EMT: Epithelial-mesenchymal transition
EVs: Extracellular vesicles
TME: Tumor microenvironment
MDR: Multidrug resistance
TACE: Transcatheter arterial chemoembolization
OS: Overall survival
tRNAs: Transfer RNAs
miRNAs: MicroRNAs
siRNAs: Small interfering RNAs
circRNAs: Circular RNAs
rRNAs: Ribosomal RNAs
lncRNAs: Long noncoding RNAs
snRNAs: Small nuclear RNAs
mRNAs: Messenger RNAs
LCSCs: Liver cancer stem cells
USP22: Ubiquitin-specific protease 22
HIF-1α: Hypoxia-inducible factor-1α
CAFs: Cancer-associated fibroblasts
HGF: Hepatocyte growth factor
ceRNA: Competing endogenous RNA
TAMs: Tumor-associated macrophages
MVs: Microvesicles; LSECs
LSECs: Liver sinusoidal endothelial cells
PD-L1: Programmed cell death ligand-1
KCs: Kupffer cells
Tregs: Regulatory T cells
DCs: Dendritic cells
MDSCs: Myeloid-derived suppressor cells
NK: Natural killer
LAG-3: Lymphocyte activation gene 3
AOSC: American Society of Clinical Oncology
GPC-3: Glypican-3
ROS: Reactive oxygen species
PUFAs: Polyunsaturated fatty acids
GSH: Glutathione
GSS: Glutathione synthetase
Glu: Glutamate
Gly: Glycine
GCL: Glutamate-cysteine ligase
NADPH: Nicotinamide adenine dinucleotide phosphate
NAD+: Nicotinamide adenine dinucleotide phosphate
AA: Arachidonic acid
AdA: Adrenic acid
ACSL4: Long-chain acyl-CoA synthetase member 4
LPCAT3: Lysophosphatidylcholine acyltransferase 3
PE: Phosphatidylethanolamines
